# Insecticide Susceptibility and Detoxification Enzyme Activity of *Frankliniella occidentalis* under Three Habitat Conditions

**DOI:** 10.3390/insects14070643

**Published:** 2023-07-17

**Authors:** Rui Fan, Zongfang Fan, Zhongxiang Sun, Yaping Chen, Furong Gui

**Affiliations:** 1 State Key Laboratory of Conservation and Utilization of Biological Resources of Yunnan, College of Plant Protection, Yunnan Agricultural University, Kunming 650201, China; ruifan1023@163.com (R.F.); szx@ynau.edu.cn (Z.S.); cyp83@ynau.edu.cn (Y.C.); 2 College of Agronomy, Xinjiang Agricultural University, Urumqi 830052, China; 18699089650@163.com

**Keywords:** *Frankliniella occidentalis*, susceptibility, insecticides, detoxification enzymes

## Abstract

**Simple Summary:**

Due to the frequent application of insecticides, resistance in field populations of *F. occidentalis* is increasing, thus complicating management efforts. However, the mechanism of resistance of *F. occidentalis* to insecticides is unclear. In order to understand the status of insecticide resistance of *F. occidentalis* in different habitats, we provide a basis for the development of resistance management strategies and study the mechanisms that confer this resistance. We evaluated the susceptibility of *F. occidentalis* to six commonly used insecticides in three field populations. The findings indicated that reduced susceptibility to the investigated insecticides of *F. occidentalis* in different habitats was due to increased activity of detoxifying enzymes. The results from our study will enrich the understanding of how invasive thrips cope with insecticide stress and shed light on how best to manage the resistance of thrips pests to insecticides in the future.

**Abstract:**

*Frankliniella occidentalis* is a highly destructive and invasive agricultural pest that has developed resistance to a variety of insecticide classes. Different planting structures and insecticide use frequency can directly affect the resistance development of *F. occidentalis*. In this study, the susceptibility of three field strains of *F. occidentalis*, collected over one year (April to November) from three habitat conditions (facility agriculture area, FA; open field crop area, OF; agroforestry intersection area, AI), to spinetoram, spinosad, emamectin benzoate, chlorfenapyr, acetamiprid, and imidacloprid were monitored and compared. At the same time, the detoxification enzyme activity of *F. occidentalis* in different habitats was determined. The results showed that the susceptibility of the *F. occidentalis* population in FA was significantly lower than that of populations from OF and AI. Among them, the *F. occidentalis* population in FA had developed low levels of resistance to spinetoram (RR = 9.18-fold), emamectin benzoate (RR = 5.47-fold), chlorfenapyr (RR = 6.67-fold), and acetamiprid (RR = 7.49-fold), and had developed moderate level resistance to imidacloprid (RR = 11.67-fold), while still being relatively sensitive to spinosad. The population of *F. occidentalis* from OF had developed low level resistance to spinetoram (RR = 5.24-fold) but was still relatively sensitive to the other five insecticides. The resistance of *F. occidentalis* from AI to six insecticides was at relatively sensitive levels. The results of the enzyme activities of detoxification enzymes, including carboxylesterase (CarE), glutathione S-transferase (GST), acetylcholinesterase (AChE), and the cytochrome P450 enzyme system (CYP450), revealed that the activities of the FA population of *F. occidentalis* were significantly higher than those of the other two populations. The change of CarE activity in *F. occidentalis* was consistent with that of spinetoram resistance, indicating that CarE may be involved in the metabolic resistance of *F. occidentalis* to spinetoram. Among the three populations, the resistance and detoxification enzyme activities of *F. occidentalis* of the FA population to six insecticides were higher than those of the other two populations. Our findings, along with other strategies, are expected to help with the resistance management of *F. occidentalis* in different habitats.

## 1. Introduction

The western flower thrips, *Frankliniella occidentalis* (Pergande) (Thysanoptera: Thripidae), which originate from North America, are among the most important invasive pests for horticultural and agronomic crops around the world [1]. *F. occidentalis* first invaded China in Beijing in 2003. Since then, it has spread rapidly to Yunnan, Zhejiang, Guizhou, and other provinces and has caused serious economic losses in China [2,3]. The host plants of *F. occidentalis* are diverse, spanning more than 600 species, including ornamental, vegetable, cotton, tobacco, legumes, shamrock, and many weeds around the planting fields [4,5], but displays different preferences for specific host plants. It causes direct damage by its feeding and oviposition, and it indirectly inflicts damage through the transmission of plant viruses, of which the tomato spotted wilt virus (TSWV) is the most economically important [6]. Therefore, due to direct and indirect damage, greenhouse producers have minimal tolerance for this insect pest.

To date, *F. occidentalis* control has been dependent on the application of insecticides [7]. Unfortunately, this reliance on insecticides has often led to the development of resistance to major insecticides, such as organophosphates, carbamates, pyrethroids, and spinosyn [8,9]. *F. occidentalis* has readily evolved resistance to a range of new insecticides in its invaded range, even in areas where it is a relatively recent invader [10]. The previous studies on this matter reported that the occurrence of resistant populations of *F. occidentalis* has been found in many countries, such as America, Australia, Spain, and China [11,12,13,14,15]. Owing to their high efficacy in controlling thrips, most growers only use certain insecticides. These rapidly become almost the only insecticide used against the thrips in certain areas, with frequent applications per host growing cycle. For example, in North Carolina, spinetoram has been used to manage *F. occidentalis* on fruiting vegetable crops for many years. However, extensive reliance on these pesticides has caused them to have poor efficacy [16,17]. At the same time, Bilbo et al. [18] found that field populations of *F. occidentalis* in North Carolina possess high levels of resistance to spinetoram. In this regard, many previous studies have also revealed the rapid development of resistance by thrips pest species to various insecticides [2,8,10,19,20]. On the other hand, resistance levels can vary widely between relatively small areas of geographic origin and areas of invasion, reflecting the unique history of insecticide exposure and selection in individual populations. For example, in China, low levels of resistance to imidacloprid have been reported from the Changping area, while moderate levels of resistance to imidacloprid have been reported from the Daxing area, although both Changping and Daxing are in Beijing [14]. Therefore, the issue of insecticide resistance in *F. occidentalis* has always been a serious threat with the capacity to lead to difficulties in the successful control of thrips.

Determining the population density and resistance development of insects in different planting environments is a prerequisite for pest forecasting and scientific prevention and control. The population dynamics of thrips are known to vary among species, host plants, and plant parts [21,22]. Studies in North Carolina have shown that *F. fusca* is abundant on winter weed species in the winter and spring [23], whereas *F. fusca* is less abundant than *F. occidentalis* on pepper and tomato plants in Georgia and North Carolina [24]. *F. occidentalis* often occurs on cultivated and wild host plants, especially on the flowers of commercially important plants such as rose *Rosa rugosa* and chrysanthemum *Chrysanthemum morifolium* [25,26]. In addition, *F. occidentalis* can occur on a variety of weeds near crops. For example, it was found to be the most dominant species among weeds in vegetable production areas in Turkey [27]. Several studies have reported on the population dynamics and biology of thrips for numerous crop and uncultivated plant species [28,29,30]. Hu et al. [31] showed that the principal occurrence period of *F. occidentalis* on *Hypericum monogynum* and clover was earlier than that of pepper and also that the primary outbreak period occurred in early May. Zhang et al. [32] pointed out that the occurrence time of *F. occidentalis* on three different host plants, including *Rosa rugosa*, *Chrysanthemum morifolium*, and *Phaseolus vulgaris*, was faster and earlier than that of the local relative *F. intonsa*. These research results provide a scientific basis for the prediction of *F. occidentalis* on different crops. At the same time, the population of *F. occidentalis* in different habitats is also affected by the types and doses of pesticides applied. A previous study reported that imidacloprid stress can increase the proportion of females in the population of *F. occidentalis* [33]. Yang et al. [34] showed that the application of spinosad is capable of accelerating the developmental duration of the immature stage of *F. occidentalis*. Kordestanie et al. [35] reported that stressing *F. occidentalis* with matrine LC_25_ can prolong the developmental duration of each stage of the F1 generation. Therefore, it is speculated that the population density of *F. occidentalis* may be related to its resistance to insecticides, resulting in less effective chemical control. 

Carboxylesterase (CarE), glutathione S-transferase (GST), acetylcholinesterase (AChE), and the cytochrome P450 enzyme system (CYP450) are important metabolic detoxifying enzymes found in insects. Detoxification enzymes in insects are induced by various exogenous or endogenous compounds, meaning that insects can quickly adapt to environmental stresses, such as insecticides [33], extreme temperatures [36] and carbon dioxide [37], etc. Research has shown that insecticides have different effects on insect defense enzyme systems, and that the changes in the activity of the enzymes are related to the toxic death of insects or indeed the development of insect resistance. Several studies have reported that insects protect themselves against insecticides such as imidacloprid by upregulating the activities of CarE, GST, and AChE. Wang et al. [38] showed that the use of the insecticides imidacloprid, diflubenzuron, and abamectin at low concentrations can promote the activity of CarE and GST in *Ambrostoma quadriimpressum*. Previous studies have shown that AChE is involved in the resistance of carbamates and organophosphate insecticides [39]. Li et al. [40] showed that CarE, GST, and CYP450 activity in *F. occidentalis* increase significantly after treatment with an LC_25_ dose of spinetoram, while the activity of AChE decreases. Detoxification enzymes play essential roles in the survival of insects exposed to adverse environments [41,42,43,44]. Therefore, higher detoxifying enzyme activity indicates the involvement of those enzymes in insecticide stress, thereby changing the insecticide susceptibility in insects.

Currently, the factors leading to decreased susceptibility of *F. occidentalis* to insecticides are unknown, but may be related to host plants, selective pressure on insecticides, or environmental conditions. The objective of this study was to evaluate the status of resistance to six insecticides in *F. occidentalis* populations collected from different hosts in three different habitats of Yunnan Province, China. At the same time, the detoxifying enzyme activities of different *F. occidentalis* populations were determined in order to clarify the adaptation mechanism of insecticides to *F. occidentalis* from the biochemical level, which could provide a theoretical basis for the rational use of insecticides in production. It also guides further research on insecticide resistance management of *F. occidentalis*.

## 2. Materials and Methods

### 2.1. Investigation of the Population Counts of F. occidentalis in the Field

In order to compare the population numbers of *F. occidentalis* in different fields, a 1-year field study was conducted in each area from 15 April to 15 November, with sampling conducted every 30 days from 15 April. The survey sites are shown in Table 1. At each location, thrips were sampled using the 5-point sampling method, with each sampling plot covering an area of approximately 100 m^2^. Five plants of each host were selected at each sample point, and six to eight flowers from each plant were randomly sampled to count the number of adults of *F. occidentalis*. When processing the thrips on a sampled site, we first beat the host plants to make the thrips fall into the white porcelain plate (30 × 22 × 4 cm). These were then collected separately into a plastic tube (50 mL) with a fine brush, marked and transferred into our laboratory for species identification, wherein the male and female adults of *F. occidentalis* were counted under stereoscopic microscopes (10× to 20×). In all study sites, the other collected species were discarded and were not included in the results of our experiment. In addition, all adult samples of *F. occidentalis* were reared on kidney beans (*Phaseolus vulgaris* (cv. Oasis)) in artificial climate chambers (ACCs; LTC-1000, SANTN, Shanghai, China) before the bioassays were determined.

### 2.2. Collection and Rearing of F. occidentalis Populations

We took susceptible strains of *F. occidentalis* as a reference to field-collected populations. This population was obtained from Kunming, Yunnan Province, China (102°46′ E, 24°54′ N), and continuously reared for 30 generations in plastic boxes (17 × 11 × 7 cm) containing kidney beans in ACCs for use in experiments. This strain was always kept in ACCs after being collected without exposure to any insecticides. Other conditions included 16/8 h light/darkness cycles and 65% relative humidity (RH). Light and dark phase temperatures were set at 27 and 25 °C.

A total of 3 *F. occidentalis* populations were collected from Yunnan Province from April to November 2021. The sampling sites are shown in Table 1. In order to assess the differences in resistance in a smaller geographical area, we tested 3 populations from the 3 regions of Yunnan Province. Crops in these areas are seriously damaged by *F. occidentalis*. The collected populations were all female adults of *F. occidentalis.* After being brought to the laboratory, they were cultured for 2 or 3 generations on kidney beans as a bioassay.

### 2.3. Toxicity Effect of Insecticides on F. occidentalis

The toxicity effect of 6 insecticides on 3 field-collected and susceptible strains of *F. occidentalis* was determined via the performance of a dipping bioassay (Table 2). Each insecticide was diluted using distilled water containing 0.1% Triton X–100 (Beijing Solar Bio Science and Technology Co. Ltd., Beijing, China) into 5 concentrations that induced thrips mortalities ranging from 5 to 99%. Culture dishes (diameter = 35 mm) and kidney bean pods (about 20 mm length) were dipped for 2 h and 15 s in different insecticide suspensions and dried at room temperature. Control treatments were dipped in distilled water containing 0.1% Triton X–100. A total of 50 random thrips were picked and placed on culture dishes sealed with cling wraps. Then, 50 holes were made using a sterilized needle for the purpose of ventilation. Female adult specimens of *F. occidentalis* were collected from the field for inclusion in the bioassay. For the purpose of maintaining consistency, only female specimens were used as they were the most abundant. Susceptible strains of *F. occidentalis* used both female and male adults 2 days after emergence, as well as 2^nd^ instar nymphs that hatched 3 to 4 days after spawning for the bioassay. The mortality rate of the thrips was assessed after 48 h. The experiments were performed in triplicate. Thrips were presumed dead when they showed no sign of antenna or leg reaction when touched using a brush. We derived and calculated the concentration–mortality regression equation and LC_50_ of insecticides against *F. occidentalis*.

### 2.4. Extraction of Enzymes from F. occidentalis

Following the method employed by Cao et al. [45], an enzyme extract of *F. occidentalis* adults was extracted and measured for the purpose of detoxifying (CarE, GST) enzyme activity. A total of 20 mature thrips were placed in micro-centrifuge tubes (Biosharp, Beijing Labgic Technology Co., Ltd., Beijing, China). Subsequently, they were added to 1200 µL phosphate-buffered saline (PBS, 0.1 mol/L, pH = 7.6), ground with a high flux tissue grinder (Tissuelyser-48, Shanghai Jingxin Industrial Development Co., Ltd., Shanghai, China) in an ice-water bath, and centrifuged at 10,000 rpm for 15 min. Subsequently, we used the supernatant as the test enzyme solution for CarE and GST.

AchE and CYP450 were extracted using ELISA kits (Sino Best Biological Technology Co., Ltd., Shanghai, China) in accordance with the manufacturer’s instructions. A total of 20 mature thrips were placed in centrifuge tubes containing 1200 µL of 0.86% physiological saline, homogenized in an ice-cold water bath using a high flux tissue grinder and centrifuged at 3000× *g* for 10 min at 4 °C using a centrifuge (Prism R, Labnet International, Inc. Edison, NJ, USA). The supernatant was used as the test enzyme solution for AchE and CYP450.

The protein content of the enzyme solution was determined by employing the bicin chonininc acid (BCA) method in accordance with the kit instructions of Nanjing Jiancheng Bioengineering Institute, Nanjing, China.

### 2.5. The Enzymatic Activity of F. occidentalis

CarE activity was determined as described by Zhang et al. [46]. The fast blue RR salt (4-benzamido-2,5-dimethoxy benzenediazoniu, a diazonium salt; 20 mg) and 100 mmol/L α-naphthyl acetate (0.2 mL) were blended with phosphate-buffered saline (PBS; pH 6.0, 0.2 mol/L, 10 mL) in order to obtain the filtered mixture. 200 µL of the mixture and 20 µL of CarE enzyme were added to a 96-well microplate and the solution was mixed immediately. Reactions were monitored continuously for 5 min at a wavelength of 450 nm (Varioskan LUX, Thermofisher, Waltham, MA, USA).

GST activity (1-chloro-2,4-dinitrobenzene, CDNB) was determined as described by Cao et al. [45]. A total of 20 µL of GST enzyme, 100 µL of CDNB (2 mmol/L), and 100 µL of reduced glutathione (GSH, 12.5 mmol/L) were mixed in a microplate. These were then left for 2 min to equilibrate. The OD was recorded continuously for 5 min at 340 nm using a microplate reader. We used (OD/min)/enzyme volumes/protein contents to represent CarE and GST activities. 

AChE and CYP450 activity were determined using ELISA kits (Sino Best Biological Technology Co., Ltd., Shanghai, China) following the manufacturer’s protocol.

### 2.6. Statistical Analysis

Data were checked for normality and homoscedasticity before statistical analyses were performed. All data were analyzed using SPSS software (version 26.0). All bioassay data were derived and calculated using log-probit analyses. The median lethal concentration (LC_50_), concentration mortality regression equations, and 95% confidence intervals (95% CIs) were calculated accordingly. Resistance ratios (RR) were calculated by dividing the LC_50_ of a field population by the corresponding LC_50_ of the susceptible strain. LC_50_ values were considered to be significantly different if their 95% CIs did not overlap [47]. The classification of resistance levels was achieved based on the RR value, RR < 5-fold as susceptibility, RR = 5 to 10-fold as low resistance, RR = 10 to 40-fold as moderate resistance, RR = 40 to 160-fold as high resistance, and >160-fold as extremely high resistance [48]. The statistical significance of differences among treatments was determined using a 1-way analysis of variance (ANOVA). The association between application frequency of insecticide per month, RR, and detoxification enzymes in *F. occidentalis* under different habitat conditions was explored via Pearson’s correlation analysis. This analysis was performed using OriginPro 2022 SR1.

## 3. Results

### 3.1. Population Counts of F. occidentalis under Different Habitat Conditions

**Figure 1:**
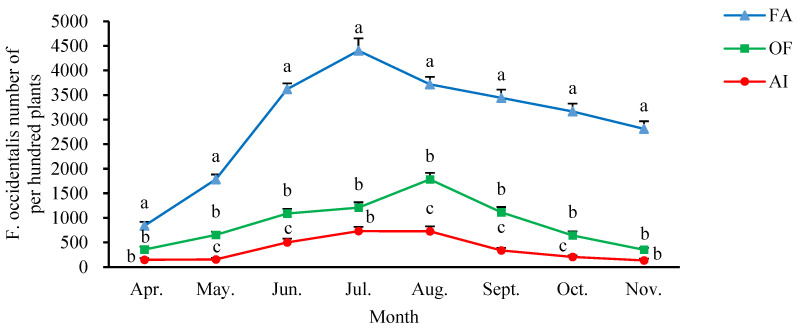
Population numbers of *F. occidentalis* in different field populations. Data are means ± *SE*. Different lowercase letters represent significant differences among different populations in the same occurrence period (LSD, *p* < 0.05). FA: facility agriculture area; OF: open field crop area; AI: agroforestry intersection area.

This study presents the population numbers of *F. occidentalis* in three different habitat conditions from April to November 2021, as shown in Figure 1. The trend indicates an initial increase followed by a decrease. The population numbers of *F. occidentalis* in different habitat conditions during the same period followed the order of FA > OF > AI. Interestingly, *F. occidentalis* showed similar distribution patterns with varying peaks under a variety of different habitat conditions. In July, the highest peaks of *F. occidentalis* were 4404.00 ± 248.09 and 732.00 ± 89.10 per hundred plants collected from FA and AI, respectively. The peak of 1784.00 ± 133.797 per hundred plants collected from OF appeared in August.

### 3.2. Toxicity Effect of Six Insecticides on Susceptible Strain of F. occidentalis

The toxicity of six insecticides to second instar nymphs, females, and males of *F. occidentalis* was found to occur in the following order: spinetoram > spinosad > emamectin benzoate > chlorfenapyr > acetamiprid > imidacloprid. Among all the insect states, the second instar nymph of *F. occidentalis* had the highest susceptibility to insecticides, followed by males and then females (Table 3). Among the tested insecticides, the female, male, and second instar nymphs of *F. occidentalis* had the highest susceptibility to spinetoram, with LC_50_ values of 0.017 mg·L^−1^, 0.014 mg·L^−1^ and 0.011 mg·L^−1^, respectively, indicating that they were the most virulent and had better toxicity. Conversely, they were the least sensitive to imidacloprid, with LC_50_ values of 1053.786 mg·L^−1^, 969.102 mg·L^−1^, and 821.241 mg·L^−1^, respectively. The toxicity of spinetoram to the second instar nymph of *F. occidentalis* was the highest, with an LC_50_ of 0.011 mg·L^−1^, which was 0.69 times that of the female and 0.90 times that of male specimens, while that of imidacloprid was the lowest, with an LC_50_ of 731.377 mg·L^−1^, which was 0.86 times of that of female and 0.87 times that of male specimens.

### 3.3. Susceptibility of F. occidentalis to Six Insecticides

The three *F. occidentalis* populations varied in susceptibility to the six insecticides tested. Based on the overlap of 95% CI from the LC_50_ ratio S/R, the susceptibility to insecticides of *F. occidentalis* in the FA population was significantly lower than that in the OF and AI populations, and that in the OF population was lower than that in the AI population, but the difference was not significant (Table S1). Among the three populations, the FA population of *F. occidentalis* developed a low resistance level to spinetoram (RR = 6.63~9.18-fold). The resistance of *F. occidentalis* to emamectin benzoate (RR = 3.98~5.47-fold) and chlorfenapyr (RR = 2.49~6.67-fold) varied from susceptible to low, but the relative susceptibility level of FA populations of *F. occidentalis* to spinosad (RR = 1.61~3.81-fold) did not. The resistance of *F. occidentalis* to acetamiprid showed an increasing trend and reached a low level (RR = 7.49-fold) in November. The resistance of *F. occidentalis* to imidacloprid ranged from susceptible to moderate resistance (RR = 10.19~11.67-fold). Furthermore, the resistance of the OF population of *F. occidentalis* to spinetoram developed from susceptible to low (RR = 2.21~5.24-fold), but the species remained susceptible to the other five insecticides (spinosad, emamectin benzoate, chlorfenapyr, acetamiprid, and imidacloprid). As a parallel, the AI population of *F. occidentalis* remained susceptible to six insecticides from April to November (Table 4 and Tables S1–S6).

### 3.4. Correlation between Different Habitat Conditions and Resistance Ratio of F. occidentalis

The correlation analysis showed that the application frequency of insecticide per month, average daily temperature, and average daily precipitation with the resistance ratio in the FA population of *F. occidentalis* to spinetoram was positively correlated but was negatively correlated with the resistance ratio to chlorfenapyr, acetamiprid, and imidacloprid. In addition, the resistance ratio in the OF population of *F. occidentalis* to insecticide (except for imidacloprid) was positively correlated with the application frequency of insecticide per month, average daily temperature, and average daily precipitation. Furthermore, the resistance ratio in the AI population of *F. occidentalis* to insecticide was positively correlated with the average daily temperature (Table 5).

### 3.5. Activity of Detoxifying Enzymes in F. occidentalis

**Figure 2:**
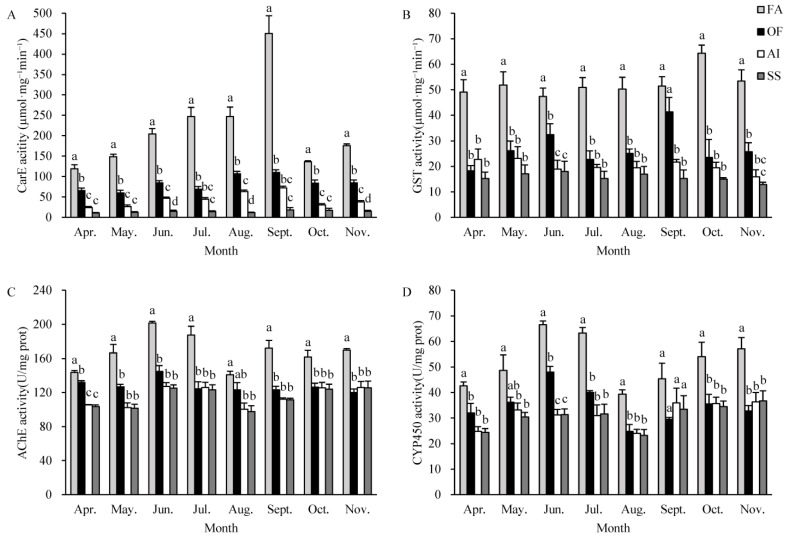
Detoxification enzyme activity in adults of *F. occidentalis* under different habitat conditions. Data in the figure are means ± SE. Different lowercase letters indicate significant differences among different habitats at the same collection time. (**A**) CarE activity; (**B**) GST activity; (**C**) AChE activity; (**D**) CYP450 activity.

In order to determine the role of detoxification enzymes in the insecticide resistance of *F. occidentalis*, enzyme assays were performed to measure the activities of CarE, GST, AChE, and CYP450. The results showed that the activity of CarE, GST, AChE, and CYP450 increased in all three habitats compared with the susceptible strain (Figure 2A–D).

The highest level of CarE activity was registered for specimens recovered from FA, followed by those from OF, and then AI. The CarE activity of *F. occidentalis* collected from FA, OF, and AI showed a trend of first increasing and then decreasing, with the highest levels of activity reached in September. CarE activity in *F. occidentalis* collected from FA, OF, and AI increased 25.26-fold, 6.18-fold, and 4.06-fold compared to the susceptible strain, respectively (Figure 2A). At the same collection time, CarE activity of *F. occidentalis* in FA was significantly higher than that of OF and AI. Furthermore, the CarE activity of the *F. occidentalis* in AI increased the least compared with that of the other two populations (Figure 2A). 

A similar trend was found with GST activity of *F. occidentalis* in three habitats, with the highest GST activity found in the FA population and the lowest in the AI population (Figure 2B). The GST activity of *F. occidentalis* collected from FA reached the highest in October, with a 4.31-fold increase compared with the susceptible strain. However, the GST activity of *F. occidentalis* collected from OF reached the highest in September, increasing 2.71-fold compared with the susceptible strain (Figure 2B). In addition, the GST activity increased the least of the *F. occidentalis* in AI compared with the other two populations (Figure 2B).

Compared with the susceptible strain, the AChE activities of *F. occidentalis* from FA were increased, but the change in OF and AI populations was not significant (Figure 2C). The AChE activity of *F. occidentalis* collected from FA reached the highest in June, increased 1.61-fold compared with the susceptible strain. However, the AChE activity of OF and AI populations showed varying degrees of change with collection time (Figure 2C).

The study found that the activity of CYP450 in *F. occidentalis* was highest in the population collected from FA (Figure 2D). The CYP450 activity in *F. occidentalis* collected from FA and AI habitats reached its peak in June, with 2.12-fold and 1.53-fold increases, respectively, compared with the susceptible strain. However, the activity of the AI population did not show significant changes (Figure 2D).

### 3.6. Correlation between Resistance Ratio and Detoxifying Enzymes in F. occidentalis under Different Habitat Conditions

**Figure 3:**
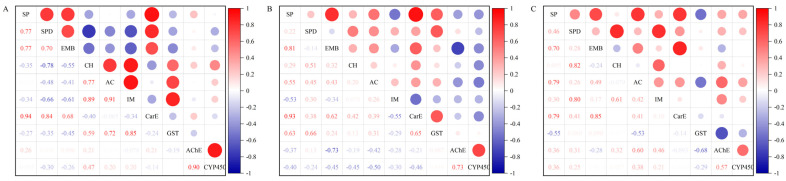
Correlation between resistance ratio (SP, spinetoram; SPD, spinosad; EMB, emamectin benzoate; CH, chlorfenapyr; AC, acetamiprid; IM, imidacloprid) and activities of detoxifying enzymes (CarE, carboxylesterase; GST, glutathione S-transferase; AChE, acetylcholinesterase; CYP450, cytochrome P450 enzyme system) in FA (**A**), OF (**B**), and AI (**C**) populations of *F. occidentalis*. Blue circles represent negative correlations and red circles represent positive correlations between six insecticides. The larger circles represent the stronger correlation. Red numbers represent positive correlation coefficients. Blue numbers represent negative correlation coefficients. Darker shades of the same color represent higher correlation coefficients.

The correlation analysis showed that the resistance ratio in the FA population of *F. occidentalis* to spinetoram and spinosad was positively correlated with CarE activity. On the other hand, the resistance ratio of *F. occidentalis* to spinetoram was positively correlated with spinosad. AChE activity was positively correlated with the activities of CYP450 (Figure 3A). Similarly, the resistance ratio in the OF population of *F. occidentalis* to insecticides was positively correlated with CarE and GST activity (Figure 3B). This result was also found in the AI population of *F. occidentalis* (Figure 3C).

## 4. Discussion

As polyphagous insects, *F. occidentalis* have many host plant species and prefer to live and feed on flowers. The density of *F. occidentalis* populations differs among host plant species [49,50]. Differences in insect population size on different host plants often reflect differences in their host plant preference. Our investigation found that the frequency of insecticide use in the FA area was much higher than that in the other two areas (Table 1), indicating that insecticides used by flower growers may also have affected thrips populations in the field [14]. Consequently, to objectively evaluate the performance of *F. occidentalis* in the field, we further studied the influence of pesticide use on the susceptibility to insecticides of *F. occidentalis* populations. The results showed that the susceptibility to insecticides is different between the populations, and that the same population owed different sensitivities to different kinds of insecticides. Among the three populations, the susceptibility of the FA population to six insecticides was the lowest, while the AI population had the highest susceptibility (Table 4). The results also showed that the decreased susceptibility of *F. occidentalis* to spinetoram and spinosad was significantly correlated with the application frequency of insecticide per month (Table 5). In other words, the frequency of pesticide use in different habitats may promote the resistance development of *F. occidentalis* populations. A study by Bielza et al. [51] showed that the 30-fold difference in the susceptibility of field populations to spinosad indicates escalating selection for resistance in fields with continuous spinosad application. The study conducted by Wang et al. [14] revealed that the Shouguang (SG) population displayed the highest levels of resistance among all the tested populations. This could be attributed to the frequent exposure of insect populations in this region to a variety of insecticides, which is consistent with our findings. Additionally, among the insecticides that we evaluated, spinetoram and spinosad showed the highest toxicity to *F. occidentalis*. Worldwide, however, farmers have reported control failures for spinosad against thrips [9,52,53,54]. Indeed, the overuse of this insecticide in greenhouses in North America and Australia, with spinosad being applied multiple times a year, has produced resistant thrips populations [12,54,55]. Our results indicate that FA and OF populations of *F. occidentalis* developed low levels of resistance to spinetoram. Langfield et al. [56] also reported a low level of resistance to spinosad in a population of *F. occidentalis* collected in Australia. Wang et al. [14] reported that 15.64-fold and 17.29-fold resistance to spinosad and spinetoram was detected in the SG population. Shen et al. [57] reported that the greatest difference among *F. occidentalis* populations was for spinetoram, with the highest LC_50_ value of a population, 73.92 times greater than that of the most susceptible population. However, studies elsewhere have also shown that levels of spinosyn resistance do not change significantly under sustained selection pressure [7]. It was suggested that spinosyn resistance may not be stable under field conditions [10,13]. Therefore, if well-designed resistance management strategies are implemented, the life span of spinosyns can be extended for the effective control of *F. occidentalis*.

The development of insect resistance is primarily regulated by the activity of detoxifying enzymes within the insects, the factors that enhance their metabolic ability to counteract pesticides. The detoxification ability of insects is reflected by the response of detoxifying enzymes to insecticides [58,59]. CarE, GST, AChE, and CYP450 are important physiological metabolic detoxification enzymes in insects. In addition, these enzymes mediate metabolic resistance to numerous pesticides [60,61]. Our results showed that the resistance of the FA population to six insecticides was the highest. Furthermore, the activities of CarE, GST, AChE, and CYP450 in *F. occidentalis* from FA were much higher than those in other populations (Figure 2). Different insecticides may activate or inhibit detoxifying enzymes in insects, providing power for the evolution of insect resistance [62,63]. It has been reported that high levels of detoxifying enzyme activity are associated with the resistance ratio in thrips strains. In this study, the resistance of *F. occidentalis* in FA and OF populations to spinetoram was significantly correlated with the application frequency of insecticide per month and CarE activity (Figure 3). Taken together, it is speculated that the increased activity of metabolic detoxification enzymes may be the reason for the decreased susceptibility to insecticides of *F. occidentalis*, a fact which has been confirmed in *Scirtothrips citri* [64]. Previous studies have found that the higher the CarE activity in *Nilaparvata lugens*, the higher the resistance to organophosphorus insecticides [65]. The increase in CarE activity in *Nilaparvata lugens* accelerated its metabolism to spirotetramat, which led to the enhancement of resistance to spirotetramat [66]. Our results are similar to those of the research described above. In the current study, the decreased susceptibility of *F. occidentalis* to spinetoram was linked to changes in CarE activity, suggesting that CarE plays a role in the detoxification metabolism of spinetoram. Previous studies showed that spinetoram treatment can increase the activities of CarE in *F. occidentalis* [37]. However, the precise mechanism by which of spinetoram stress induces increased activity of CarE should be explored further.

Overall, the degree of insecticide application in different areas is closely related to the susceptibility of pests to insecticides, and the improper use of chemical insecticides will lead to the emergence of resistance of pests. The effects of different insecticides on the detoxification enzyme activity of *F. occidentalis* are different. *F. occidentalis* can adapt to the stress pressure from pesticides by regulating the activity of detoxification enzymes in their bodies to enhance their adaptability to the insecticide environment. Nevertheless, there are limitations to our study. Although our study revealed among-population variation in susceptibility to several insecticides in *F. occidentalis*, we were not able to reflect the variation in resistance over the years. Therefore, we have focused on activities related to detoxifying enzymes among populations to different insecticides, which has highlighted a mechanism leading to resistance in the field. The rapid development of insecticide resistance in pest thrips emphasizes the importance of resistance monitoring and management. Therefore, it is necessary to develop effective management plans to delay any resistance development.

## 5. Conclusions

In conclusion, we found that the susceptibility of the FA population of *F. occidentalis* was significantly lower than that of the other two populations. The FA population of *F. occidentalis* developed low-level resistance to spinetoram, emamectin benzoate, chlorfenapyr, and acetamiprid and moderate resistance to imidacloprid. In addition, the activities of CarE, GST, AChE, and CYP450 in FA *F. occidentalis* were highest. The change in CarE activity in *F. occidentalis* was consistent with that of susceptibility to spinetoram, indicating that CarE may be involved in the metabolic resistance of *F. occidentalis* to spinetoram. Therefore, in the control of *F. occidentalis*, different insecticides should be selected according to the susceptibility to insecticides of *F. occidentalis* populations in different habitats in order to improve the control effect.

## Figures and Tables

**Table 1:** Detailed information on field populations of *F. occidentalis* in 3 habitats.

Code of Field Population	Location of Collecting Sites	Longitude (E) and Latitude (N)	Host Plant	Average Daily Temperature (°C)	Average Daily Precipitation (mm)	Application Frequency of Insecticide per Month	Common Use Insecticide
Facility agriculture area (FA)	Chenggong District of Kunming	102°46′56″ E, 24°54′15″ N	Rose	18.13 ± 1.17	3.4 ± 1.13	7.50 ± 0.60 a	60 g/L Spinetoram SC 70% Imidacloprid WP 10% Chlorfenapyr SC 1.8% Abamectin EC 20% Methomyl AS
Open field crop area (OF)	Zhaoyang District of Zhaotong	103°39′57″ E, 27°14′8″ N	Tobacco, Weed, Rape flowers	16.74 ± 1.75	5.85 ± 1.85	5.50 ± 0.60 b	
Agroforestry intersection area (AI)	Panlong District of Kunming	102°44′32″ E, 25°07′45″ N	Shamrock	18.13 ± 1.17	3.4 ± 1.13	1.25 ± 0.16 c	

SC, suspension concentrate; WP, wettable powder; EC, emulsifiable concentrate; AS, aqueous solution. Data are means ± SE. Different lowercase letters represent significant differences among different populations (LSD, *p* < 0.05).

**Table 2:** Insecticides used in bioassays listed by common name, formulation, and supplier.

Active Ingredient Common Name	Formulation	IRAC Class	Supplier
Spinetoram	60 g/L SC	5, Spinosyn	Dow AgroSciences Company, Beijing, China
Spinosad	8% WE	5, Spinosyn	Shenzhen Noposion Agrochenicals Co. Ltd., Shenzhen, China
Emamectin benzoate	77.46%	6, Avermectins	Qingdao Ruifengcote Chemical Co. Ltd., Qingdao, China
Chlorfenapyr	98%	13, Pyrroles	Qingdao Ruifengcote Chemical Co. Ltd., Qingdao, China
Acetamiprid	97%	4A, Neonicotinoid	Qingdao Ruifengcote Chemical Co. Ltd., Qingdao, China
Imidacloprid	70% WG	4A, Neonicotinoid	Jiangxi Zhengbang Crop Protection Co. Ltd., Nanchang, China

IRAC, Insecticide Resistance Action Committee. https://irac-online.org/, accessed on 23 September 2022. SC, suspension concentrate; WE, water emulsion; WG, water-dispersible granule.

**Table 3:** The toxicity of six insecticides on susceptible strain of *F. occidentalis*.

Insecticide	Developmental Stage	Concentration–Response Regression Equation	LC_50_ (mg·L^−1^) 95% CI	Correlation Coefficient	χ^2^
Spinetoram	Female	y = 3.553x + 11.325	0.017 (0.014~0.020)	0.9993	0.0688
	Male	y = 3.072x + 10.733	0.014 (0.011~0.016)	0.9809	1.6571
	Second instar nymph	y = 3.174x + 11.237	0.011 (0.009~0.013)	0.9748	2.0188
Spinosad	Female	y = 2.502x + 5.883	0.444 (0.354~0.568)	0.9973	0.1538
	Male	y = 2.758x − 6.480	0.291 (0.238~0.376)	0.9985	0.0935
	Second instar nymph	y = 2.695x + 6.540	0.268 (0.215~0.337)	1.0000	0.0007
Emamectin benzoate	Female	y = 2.187x − 5.732	2.161 (1.687~2.847)	0.9965	0.2189
	Male	y = 2.976x − 5.460	1.428 (1.152~1.737)	0.9993	0.0518
	Second instar nymph	y = 2.145x − 5.181	1.215 (0.938~1.776)	0.9978	0.0844
Chlorfenapyr	Female	y = 3.224x − 8.658	13.629 (11.614~17.076)	0.9729	1.0216
	Male	y = 4.836x − 8.614	5.588 (4.771~6.413)	0.9569	1.7674
	Second instar nymph	y = 1.945x − 6.225	4.263 (3.136~5.918)	0.9994	0.0295
Acetamiprid	Female	y = 3.230x − 11.305	89.564 (75.237~107.417)	0.9980	0.1895
	Male	y = 2.452x − 9.036	44.311 (33.715~55.667)	0.9987	0.0702
	Second instar nymph	y = 3.031x − 9.738	36.542 (29.466~44.275)	0.9762	1.9262
Imidacloprid	Female	y = 2.918x − 13.820	1053.786 (809.074~1276.846)	0.9847	1.1732
	Male	y = 2.334x − 11.971	969.102 (760.427~302.762)	0.9899	0.4908
	Second instar nymph	y = 3.278x − 14.553	821.241 (677.642~988.754)	0.9987	0.1130

LC_50_, the concentration of insecticide which is lethal to 50% of the thrips; 95%CI, 95% confident intervals. The same below.

**Table 4:** Susceptibility of field populations and a laboratory population of *F. occidentalis* to six insecticides.

Month	Population	Spinetoram		Spinosad		Emamectin Benzoate		Chlorfenapyr		Acetamiprid		Imidacloprid	
		LC_50_	RR	LC_50_	RR	LC_50_	RR	LC_50_	RR	LC_50_	RR	LC_50_	RR
Apr.	FA	0.13	6.63	0.92	2.47	7.60	3.98	29.10	2.69	201.17	2.20	4109.09	3.84
	OF	0.04	2.21	0.75	2.02	2.67	1.40	38.24	3.53	118.84	1.30	2391.67	2.24
	AI	0.04	2.26	0.52	1.42	2.23	1.17	24.82	2.29	100.19	1.10	1223.56	1.14
	SS	0.02	1.00	0.37	1.00	1.91	1.00	10.82	1.00	91.31	1.00	1069.08	1.00
May.	FA	0.13	7.11	0.87	2.45	9.10	4.90	30.34	2.69	259.26	3.15	4294.34	4.20
	OF	0.06	3.06	0.75	2.12	3.40	1.83	31.72	2.82	146.81	1.78	2503.65	2.45
	AI	0.05	2.83	0.54	1.53	3.14	1.69	25.17	2.23	103.42	1.26	1263.69	1.24
	SS	0.02	1.00	0.36	1.00	1.86	1.00	11.26	1.00	82.33	1.00	1022.45	1.00
Jun.	FA	0.13	7.88	0.75	2.47	10.91	5.01	43.12	4.75	213.63	2.71	3874.02	3.79
	OF	0.06	3.76	0.60	1.99	3.61	1.66	26.88	2.96	109.47	1.39	1472.74	1.44
	AI	0.06	3.41	0.57	1.87	3.38	1.55	26.00	2.87	107.17	1.36	1399.95	1.37
	SS	0.02	1.00	0.30	1.00	2.18	1.00	9.07	1.00	78.78	1.00	1021.19	1.00
Jul.	FA	0.14	8.18	1.16	3.04	7.78	4.48	30.07	2.55	304.53	4.21	4177.69	4.11
	OF	0.06	3.47	0.64	1.68	4.60	2.65	42.73	3.63	105.55	1.46	1943.87	1.91
	AI	0.06	3.29	0.58	1.53	3.22	1.85	24.64	2.09	108.64	1.50	1329.73	1.31
	SS	0.02	1.00	0.38	1.00	1.74	1.00	11.78	1.00	72.34	1.00	1017.65	1.00
Aug.	FA	0.14	8.47	1.20	2.93	9.61	5.47	29.07	2.56	309.74	4.00	4475.98	3.96
	OF	0.09	5.18	0.83	2.04	4.94	2.81	41.96	3.70	142.62	1.84	1984.93	1.75
	AI	0.06	3.47	0.59	1.45	3.60	2.05	24.24	2.14	105.00	1.35	1271.22	1.12
	SS	0.02	1.00	0.41	1.00	1.76	1.00	11.35	1.00	77.50	1.00	1131.33	1.00
Sept.	FA	0.16	9.18	1.34	3.81	9.73	5.38	27.69	2.49	323.03	4.08	4718.61	4.19
	OF	0.09	5.24	0.94	2.67	4.90	2.71	42.12	3.79	132.13	1.67	2181.23	1.94
	AI	0.06	3.53	0.60	1.71	3.84	2.12	25.39	2.29	105.96	1.34	1377.96	1.22
	SS	0.02	1.00	0.35	1.00	1.81	1.00	11.10	1.00	79.15	1.00	1125.54	1.00
Oct.	FA	0.14	7.11	0.80	2.09	7.07	4.03	68.90	6.39	494.51	6.80	12481.26	11.67
	OF	0.08	4.26	0.57	1.48	4.65	2.64	33.99	3.15	109.32	1.50	1916.72	1.79
	AI	0.06	3.11	0.58	1.50	2.35	1.34	24.95	2.31	101.28	1.39	1241.94	1.16
	SS	0.02	1.00	0.38	1.00	1.76	1.00	10.78	1.00	72.74	1.00	1069.68	1.00
Nov.	FA	0.13	7.47	0.61	1.61	7.88	4.05	73.81	6.67	576.22	7.49	11020.07	10.19
	OF	0.07	4.12	0.54	1.42	4.98	2.56	29.67	2.68	108.84	1.41	1830.64	1.69
	AI	0.06	3.29	0.54	1.41	2.79	1.44	23.84	2.15	102.74	1.34	1248.09	1.15
	SS	0.02	1.00	0.38	1.00	1.94	1.00	11.07	1.00	76.94	1.00	1081.00	1.00

FA, facility agriculture area; OF, open field crop area; AI, agroforestry intersection area. Resistance ratio (RR), calculated by LC_50_ of field population/LC_50_ of susceptible strain (SS). The same below.

**Table 5:** Correlation between different habitat conditions and insecticide resistance ratio of *F. occidentalis*.

Population	Conditions	Resistance Ratio (RR)					
		Spinetoram	Spinosad	Emamectin Benzoate	Chlorfenapyr	Acetamiprid	Imidacloprid
FA	AF	0.51	0.57	0.69	−0.50	−0.61	−0.71
	T	0.34	0.69	0.68	−0.81 *	−0.82 *	−0.84 **
	P	0.3	0.16	0.41	−0.036	−0.41	−0.41
OF	AF	0.34	0.46	0.25	0.54	0.63	−0.03
	T	0.22	0.61	0.058	0.61	0.45	0.0001
	P	0.24	0.11	0.39	0.63	0.12	−0.26
AI	AF	0.34	−0.24	0.55	−0.46	0.52	−0.00025
	T	0.045	0.46	0.52	0.23	0.041	0.48
	P	0.42	0.76 *	0.16	0.76*	0.35	0.73 *

AF, Application frequency of insecticide per month; T, average daily temperature; P, average daily precipitation. * *p* ≤ 0.05; ** *p* ≤ 0.01.

## Data Availability

The raw data supporting the conclusions of this article will be made available by the authors, without undue reservation, to any qualified researcher.

## Supplementary Materials

The following supporting information can be downloaded at: https://www.mdpi.com/article/10.3390/insects14070643/s1, Table S1. Susceptibility of field populations and a laboratory population of *F. occidentalis* to spinetoram; Table S2. Susceptibility of field populations and a laboratory population of *F. occidentalis* to spinosad; Table S3. Susceptibility of field populations and a laboratory population of *F. occidentalis* to emamectin benzoate; Table S4. Susceptibility of field populations and a laboratory population of *F. occidentalis* to chlorfenapyr; Table S5. Susceptibility of field populations and a laboratory population of *F. occidentalis* to acetamiprid; Table S6. Susceptibility of field populations and a laboratory population of *F. occidentalis* to imidacloprid.
