# Supplementary material for: Insecticide Susceptibility and Detoxification Enzyme Activity of Frankliniella occidentalis under Three Habitat Conditions
Source: Insects. 2023 Jul 17;14(7):643. doi: 10.3390/insects14070643 (PMC10380546; doi:10.3390/insects14070643)
Supplement: Supplementary file 1 [file insects-14-00643-s001.zip › insects-2415644-supplementary.pdf]

Supplementary material

Table S1. Susceptibility of field populations and a laboratory population of *F. occidentalis* to spinetoram

Month	Population[a]	Concentration-response regression equation	LC_{50} ($mg \cdot L^{-1}$)	95% CI ($mg \cdot L^{-1}$)	Correlation coefficient	χ^2	RR[b]
4	FA	y=1.771x+6.591	0.126	0.087~0.176	0.9980	0.2724	6.63
	OF	y=2.435x+8.353	0.042	0.026~0.054	0.9943	0.2531	2.21
	AI	y=3.310x+9.528	0.043	0.036~0.051	0.9906	0.9501	2.26
	SS	y=3.570x+11.185	0.019	0.015~0.022	0.9901	1.0011	1.00
5	FA	y=2.243x+7.003	0.128	0.099~0.171	0.9827	1.2478	7.11
	OF	y=3.235x+9.074	0.055	0.044~0.065	0.9867	1.2021	3.06
	AI	y=3.668x+9.753	0.051	0.043~0.060	0.9897	1.1390	2.83
	SS	y=3.051x+10.310	0.018	0.015~0.022	0.9973	0.2216	1.00
6	FA	y=2.713x+7.372	0.134	0.107~0.167	0.9798	1.2778	7.88
	OF	y=3.066x+8.667	0.064	0.051~0.076	1.0000	0.0025	3.76
	AI	y=3.830x+9.729	0.058	0.049~0.069	0.9905	1.0829	3.41
	SS	y=3.525x+11.272	0.017	0.014~0.020	0.9904	1.0589	1.00
7	FA	y=2.649x+7.271	0.139	0.110~0.174	0.9850	1.5669	8.18
	OF	y=2.816x+3.454	0.059	0.046~0.072	0.9884	0.8437	3.47
	AI	y=3.543x+9.425	0.056	0.048~0.066	0.9979	0.2336	3.29
	SS	y=2.837x+9.994	0.017	0.014~0.021	0.9997	0.0219	1.00
8	FA	y=2.378x+7.001	0.144	0.110~0.185	0.9923	0.8475	8.47
	OF	y=2.690x+7.843	0.088	0.070~0.111	0.9956	0.2768	5.18
	AI	y=3.214x+8.942	0.059	0.050~0.071	0.9991	0.0877	3.47
	SS	y=3.020x+10.364	0.017	0.014~0.020	0.9951	0.4189	1.00
9	FA	y=2.644x+7.131	0.156	0.124~0.196	0.9927	0.7445	9.18
	OF	y=3.003x+8.158	0.089	0.073~0.109	0.9971	0.2083	5.24
	AI	y=3.642x+8.451	0.060	0.051~0.071	0.9999	0.0072	3.53
	SS	y=2.958x+10.226	0.017	0.014~0.021	0.9881	0.9543	1.00
10	FA	y=3.856x+8.356	0.135	0.114~0.158	0.9938	0.7402	7.11
	OF	y=3.070x+8.351	0.081	0.067~0.098	0.9945	0.4849	4.26
	AI	y=3.661x+8.505	0.059	0.050~0.069	0.9955	0.4995	3.11
	SS	y=3.897x+11.746	0.019	0.014~0.021	0.9863	1.7063	1.00
11	FA	y=3.216x+7.883	0.127	0.107~0.153	0.9846	1.4678	7.47
	OF	y=3.278x+8.786	0.070	0.058~0.083	0.9987	0.1271	4.12
	AI	y=3.693x+8.628	0.056	0.047~0.066	0.9940	0.6752	3.29
	SS	y=3.427x+11.062	0.017	0.014~0.020	0.9977	0.2250	1.00

Note: [a] See Table 1 for population codes; [b] The resistance ratio (RR), calculated by LC_{50} of field population/LC_{50} of susceptible strain (SS). The same below.

Table S2. Susceptibility of field populations and a laboratory population of *F. occidentalis* to spinosad

Month	Population	Concentration-response regression equation	LC_{50} ($mg \cdot L^{-1}$)	95% CI ($mg \cdot L^{-1}$)	Correlation coefficient	χ^2	RR
4	FA	y=1.977x+5.076	0.915	0.658~1.217	0.9836	1.4704	2.47
	OF	y=2.061x+5.261	0.747	0.557~0.979	0.9965	0.3408	2.02
	AI	y=4.201x+6.179	0.524	0.440~0.597	0.9831	1.1929	1.42
	SS	y=2.299x+5.994	0.370	0.278~0.472	0.9959	0.1937	1.00
5	FA	y=1.727x+5.104	0.871	0.597~1.187	0.9966	0.2292	2.45
	OF	y=2.494x+5.305	0.754	0.590~0.955	0.9953	0.6204	2.12
	AI	y=3.356x+5.887	0.544	0.456~0.654	0.9863	1.2742	1.53
	SS	y=2.757x+6.236	0.356	0.283~0.435	0.9946	0.3670	1.00
6	FA	y=2.322x+5.293	0.748	0.566~0.962	0.9954	0.5057	2.47
	OF	y=2.186x+5.481	0.602	0.445~0.779	0.9976	0.2439	1.99
	AI	y=4.073x+6.002	0.568	0.484~0.660	0.9903	1.2721	1.87
	SS	y=3.994x+7.073	0.303	0.256~0.351	0.9862	1.6483	1.00
7	FA	y=1.760x-5.111	1.156	0.840~1.659	0.9960	0.2764	3.04
	OF	y=1.803x+5.351	0.639	0.438~0.867	0.9942	0.4261	1.68
	AI	y=3.702x+5.875	0.580	0.495~0.680	0.9844	1.9193	1.53
	SS	y=3.081x+6.294	0.380	0.315~0.457	0.9877	1.1052	1.00
8	FA	y=1.933x-5.151	1.197	0.877~1.686	0.9954	0.3217	2.93
	OF	y=1.781x+5.143	0.832	0.582~1.151	0.9995	0.0326	2.04
	AI	y=3.070x+5.698	0.593	0.492~0.713	0.9945	0.4849	1.45
	SS	y=3.161x+6.232	0.408	0.337~0.491	0.9996	0.0319	1.00
9	FA	y=1.771x-5.225	1.339	0.964~1.996	0.9988	0.0743	3.81
	OF	y=1.891x+5.053	0.937	0.675~1.291	0.9827	1.2980	2.67
	AI	y=3.483x+5.776	0.599	0.502~0.710	0.9983	0.1735	1.71
	SS	y=3.063x+6.392	0.351	0.285~0.422	1.0000	0.0001	1.00
10	FA	y=3.422x-5.333	0.799	0.632~0.984	0.9963	0.5600	2.09
	OF	y=1.768x+5.434	0.568	0.412~0.781	0.9800	1.5456	1.48
	AI	y=3.686x+5.882	0.576	0.487~0.678	0.9861	1.5858	1.50
	SS	y=3.775x+6.574	0.383	0.321~0.450	0.9999	0.0142	1.00
11	FA	y=1.752x+5.371	0.614	0.419~0.834	0.9998	0.0214	1.61
	OF	y=2.137x+5.570	0.541	0.411~0.706	0.9927	0.7680	1.42
	AI	y=3.744x+6.007	0.538	0.454~0.631	0.9866	1.5795	1.41
	SS	y=2.938x+6.232	0.381	0.313~0.460	0.9952	0.3946	1.00

Table S3. Susceptibility of field populations and a laboratory population of *F. occidentalis* to emamectin benzoate

Month	Population	Concentration-response regression equation	LC_{50} ($mg{\cdot}L^{-1}$)	95% CI ($mg{\cdot}L^{-1}$)	Correlation coefficient	χ^2	RR
4	FA	y=2.793x-7.460	7.601	5.904~9.429	0.9841	1.7291	3.98
	OF	y=1.925x-5.821	2.668	1.887~3.706	0.9994	0.0933	1.40
	AI	y=3.400x-6.184	2.230	1.891~2.667	0.9959	0.4155	1.17
	SS	y=3.426x-5.964	1.912	1.581~2.276	0.9998	0.0210	1.00
5	FA	y=1.833x-6.758	9.097	6.584~12.232	0.9967	0.1895	4.90
	OF	y=1.384x-5.669	3.403	2.016~4.614	0.9971	0.2749	1.83
	AI	y=2.315x-6.152	3.144	2.205~4.096	0.9907	0.8455	1.69
	SS	y=3.187x-5.856	1.857	1.529~2.223	0.9989	0.0982	1.00
6	FA	y=3.961x-9.110	10.907	9.119~12.934	0.9955	0.6313	5.01
	OF	y=3.094x-6.726	3.614	2.873~4.318	0.9912	0.7586	1.66
	AI	y=1.436x-5.759	3.375	2.227~5.138	0.9951	0.4567	1.55
	SS	y=2.809x-5.949	2.177	1.792~2.689	0.9992	0.0624	1.00
7	FA	y=1.414x-6.260	7.780	4.708~11.223	0.9977	0.0778	4.48
	OF	y=3.094x-7.049	4.596	3.797~5.502	0.9960	0.3538	2.65
	AI	y=1.438x-5.731	3.22	2.122~4.891	0.9830	1.6204	1.85
	SS	y=2.837x-5.679	1.736	1.397~2.100	0.9997	0.0219	1.00
8	FA	y=2.137x-7.100	9.605	7.156~12.622	1.0000	0.0025	5.47
	OF	y=3.366x-7.335	4.938	4.160~5.861	0.9950	0.5158	2.81
	AI	y=1.495x-5.833	3.604	2.359~5.485	0.9962	0.3429	2.05
	SS	y=3.063x-5.748	1.755	1.425~2.109	1.0000	0.0001	1.00
9	FA	y=2.290x-7.263	9.725	7.481~12.522	0.9959	0.3313	5.38
	OF	y=3.661x-7.527	4.900	4.135~5.772	0.9964	0.3922	2.71
	AI	y=1.284x-5.750	3.838	2.402~6.247	0.9984	0.1155	2.12
	SS	y=3.875x-5.996	1.807	1.530~2.109	1.0000	0.0002	1.00
10	FA	y=2.719x-7.310	7.074	5.473~8.761	1.0000	0.0001	4.03
	OF	y=3.898x-7.600	4.645	3.942~5.426	0.9863	1.7063	2.64
	AI	y=1.340x-5.498	2.351	1.508~3.558	0.9848	1.4101	1.34
	SS	y=3.604x-5.882	1.757	1.483~2.058	0.9982	0.2009	1.00
11	FA	y=2.949x-7.643	7.879	6.366~9.598	0.9884	1.4840	4.05
	OF	y=3.664x-7.555	4.981	4.206~5.872	0.9946	0.8025	2.56
	AI	y=1.387x-5.618	2.791	1.841~4.209	0.9918	0.7917	1.44
	SS	y=3.696x-6.067	1.944	1.658~2.278	0.9977	0.2754	1.00

Table S4. Susceptibility of field populations and a laboratory population of *F. occidentalis* to chlorfenapyr

Month	Population	Concentration-response regression equation	LC_{50} ($mg \cdot L^{-1}$)	95% CI ($mg \cdot L^{-1}$)	Correlation coefficient	χ^2	RR
4	FA	y=1.554x-7.276	29.102	20.138~44.027	0.9890	0.9293	2.69
	OF	y=2.383x+6.197	38.244	28.663~50.389	0.9901	1.5418	3.53
	AI	y=1.856x-7.588	24.820	18.221~35.509	0.9938	0.3253	2.29
	SS	y=8.130x-13.410	10.824	9.884~11.743	0.9957	0.5744	1.00
5	FA	y=2.475x-8.668	30.336	23.960~39.414	0.9867	1.1221	2.69
	OF	y=0.929x-6.395	31.723	15.773~56.442	0.9945	0.1935	2.82
	AI	y=3.801x-10.325	25.171	21.148~29.660	0.9974	0.2833	2.23
	SS	y=5.752x-11.050	11.264	10.090~12.450	0.9991	0.0914	1.00
6	FA	y=1.551x-7.535	43.121	30.333~81.038	0.9877	0.4792	4.75
	OF	y=2.014x-7.879	26.881	19.188~36.231	0.9983	0.1973	2.96
	AI	y=3.214x-9.548	26.001	21.827~31.200	0.9899	0.9637	2.87
	SS	y=3.405x-8.261	9.072	7.608~10.709	0.9982	0.1818	1.00
7	FA	y=1.815x-7.683	30.069	22.223~43.301	0.9898	0.5229	2.55
	OF	y=1.589x-7.592	42.730	29.415~62.180	0.9915	0.7400	3.63
	AI	y=3.216x-9.476	24.638	20.623~29.449	0.9871	1.2478	2.09
	SS	y=5.504x-10.897	11.784	10.612~13.068	0.9918	0.7917	1.00
8	FA	y=1.763x-7.581	29.073	20.993~42.360	0.9970	0.1388	2.56
	OF	y=2.211x-8.588	41.959	30.493~56.408	0.9993	0.0933	3.70
	AI	y=3.531x-9.889	24.242	20.547~28.567	0.9923	0.8651	2.14
	SS	y=5.492x-10.795	11.354	10.132~12.602	0.9909	0.8290	1.00
9	FA	y=1.797x-7.591	27.687	20.289~39.179	1.0000	0.0002	2.49
	OF	y=1.568x-7.547	42.123	28.284~62.012	0.9931	0.5499	3.79
	AI	y=3.174x-9.458	25.393	21.041~30.590	0.9954	0.4008	2.29
	SS	y=5.276x-10.515	11.100	9.837~12.353	0.9918	0.6953	1.00
10	FA	y=1.699x-8.123	68.896	46.183~179.911	0.9990	0.0350	6.39
	OF	y=1.960x-8.002	33.985	24.977~45.910	0.9888	1.4829	3.15
	AI	y=3.483x-9.867	24.951	20.934~29.601	0.9983	0.1735	2.31
	SS	y=4.933x-10.094	10.780	9.487~12.028	0.9872	1.0290	1.00
11	FA	y=1.938x-8.620	73.810	55.306~99.697	0.9962	0.3302	6.67
	OF	y=1.823x-7.683	29.666	21.162~40.591	0.9896	1.2179	2.68
	AI	y=3.463x-9.770	23.844	19.746~28.373	0.9951	0.4710	2.15
	SS	y=5.633x-10.882	11.070	9.938~12.224	0.9859	1.4367	1.00

Table S5. Susceptibility of field populations and a laboratory population of *F. occidentalis* to acetamiprid

Month	Population	Concentration-response regression equation	LC_{50} ($mg\cdot L^{-1}$)	95% CI ($mg\cdot L^{-1}$)	Correlation coefficient	χ^2	RR
4	FA	Y=2.644x-11.091	201.165	151.469~251.806	0.9912	0.8393	2.20
	OF	Y=2.734x-10.673	118.842	96.030~151.278	0.9975	0.1550	1.30
	AI	Y=2.686x-10.375	100.193	81.916~125.394	0.9999	0.0099	1.10
	SS	Y=3.265x-11.402	91.310	75.575~110.389	0.9956	0.3787	1.00
5	FA	Y=2.038x-9.919	259.261	193.087~338.285	0.9931	0.4840	3.15
	OF	Y=2.328x-10.043	146.813	11.077~193.490	0.9990	0.0510	1.78
	AI	Y=2.465x-9.965	103.419	81.760~134.896	0.9891	0.5559	1.26
	SS	Y=2.880x-10.517	82.331	66.883~100.511	0.9996	0.0281	1.00
6	FA	Y=1.851x-9.312	213.627	144.053~285.515	0.9998	0.0113	2.71
	OF	Y=3.897x-12.947	109.466	92.498~127.552	0.9982	0.2092	1.39
	AI	Y=3.702x-12.516	107.165	91.741~126.207	0.9914	1.0137	1.36
	SS	Y=3.230x-11.125	78.782	65.688~93.785	0.9980	0.1895	1.00
7	FA	Y=1.565x-8.888	304.528	212.293~439.717	0.9997	0.0141	4.21
	OF	Y=2.921x-10.911	105.554	84.239~127.568	0.9892	0.7983	1.46
	AI	Y=3.070x-11.251	108.638	90.229~130.762	0.9945	0.4849	1.50
	SS	Y=3.461x-11.436	72.343	60.549~85.120	0.9966	0.3634	1.00
8	FA	Y=2.502x-11.233	309.743	240.452~395.571	0.9857	0.8257	4.00
	OF	Y=2.717x-10.852	142.615	114.868~180.515	0.9999	0.0045	1.84
	AI	Y=3.537x-12.148	105.001	88.914~123.597	0.9955	0.4995	1.35
	SS	Y=3.236x-11.114	77.495	64.522~92.145	0.9995	0.0431	1.00
9	FA	Y=2.607x-11.541	323.033	252.532~408.235	0.9971	0.2733	4.08
	OF	Y=2.307x-9.894	132.133	102.057~172.438	0.9991	0.0410	1.67
	AI	Y=3.328x-11.740	105.959	88.125~126.275	0.9955	0.4995	1.34
	SS	Y=2.894x-10.493	79.146	64.098~96.300	0.9966	0.2559	1.00
10	FA	Y=2.120x-10.712	494.507	360.589~653.193	0.9889	1.0394	6.80
	OF	Y=3.627x-12.394	109.323	92.410~128.007	0.9853	1.7460	1.50
	AI	Y=3.565x-12.149	101.276	85.698~118.977	0.9892	1.2404	1.39
	SS	Y=3.606x-11.714	72.740	60.791~85.555	0.9871	1.4260	1.00
11	FA	Y=1.937x-10.347	576.220	427.764~770.612	0.9986	0.1187	7.49
	OF	Y=3.134x-11.383	108.838	89.604~129.788	0.9941	0.5379	1.41
	AI	Y=3.238x-12.514	102.736	85.757~122.318	0.9840	1.5911	1.34
	SS	Y=2.956x-10.576	76.938	63.030~92.757	0.9887	0.9519	1.00

Table S6. Susceptibility of field populations and a laboratory population of *F. occidentalis* to imidacloprid

Month	Population	Concentration-response regression equation	LC_{50} ($mg·L^{-1}$)	95% CI ($mg·L^{-1}$)	Correlation coefficient	χ^2	RR
4	FA	Y=1.610x-10.820	4109.092	2889.596~6020.722	0.9975	0.1076	3.84
	OF	Y=1.196x-9.042	2391.667	1389.531~3811.654	0.9967	0.1135	2.24
	AI	Y=2.405x-12.425	1223.555	912.042~1521.568	0.9842	0.9163	1.14
	SS	Y=3.668x-11.111	1069.082	888.967~1259.980	0.9994	0.0667	1.00
5	FA	Y=1.527x-10.547	4294.344	2882.881~6691.793	0.9906	0.3217	4.20
	OF	Y=2.034x-11.914	2503.651	1881.051~3306.946	0.9954	0.4391	2.45
	AI	Y=2.900x-13.994	1263.692	996.567~1529.022	0.9801	1.5717	1.24
	SS	Y=3.622x-10.902	1022.450	853.732~1201.494	0.9913	0.9517	1.00
6	FA	Y=2.615x-14.384	3874.017	3032.665~4901.150	0.9941	0.5542	3.79
	OF	Y=1.744x-10.526	1472.744	926.588~2029.198	0.9984	0.1056	1.44
	AI	Y=3.446x-15.842	1399.948	1173.053~1648.804	0.9936	0.6834	1.37
	SS	Y=4.053x-12.197	1021.193	874.879~1181.125	0.9914	1.1473	1.00
7	FA	Y=1.982x-12.177	4177.689	3135.541~5667.350	0.9990	0.0594	4.11
	OF	Y=1.870x-11.151	1943.866	1353.767~2614.157	0.9960	0.2999	1.91
	AI	Y=3.525x-11.012	1329.731	1112.940~1560.672	0.9904	1.0589	1.31
	SS	Y=3.666x-11.025	1017.653	858.793~1189.249	0.9933	0.7828	1.00
8	FA	Y=1.222x-9.463	4475.975	2777.122~8299.559	0.9999	0.0020	3.96
	OF	Y=1.762x-10.811	1984.933	1334.173~2731.381	0.9860	0.9720	1.75
	AI	Y=3.812x-11.833	1271.215	1072.609~1478.773	0.9927	0.8645	1.12
	SS	Y=3.707x-11.320	1131.332	962.762~1322.758	0.9999	0.0065	1.00
9	FA	Y=2.895x-15.636	4718.614	3806.433~5851.487	0.9996	0.0378	4.19
	OF	Y=2.354x-12.858	2181.227	1631.093~2821.897	0.9923	0.8370	1.94
	AI	Y=3.461x-10.866	1377.955	1153.305~1621.329	0.9966	0.3634	1.22
	SS	Y=3.707x-11.320	1125.541	958.950~1316.240	0.9927	0.8944	1.00
10	FA	Y=1.347x-10.518	12481.257	7555.979~89896.753	0.9603	0.8979	11.67
	OF	Y=2.054x-11.743	1916.715	1433.457~2517.242	0.9927	0.9032	1.79
	AI	Y=3.465x-10.721	1241.942	1026.329~1460.436	0.9976	0.2439	1.16
	SS	Y=3.776x-11.440	1069.675	910.629~1247.215	0.9891	1.3620	1.00
11	FA	Y=2.436x-14.846	11020.071	8827.977~14091.660	0.9867	0.7902	10.19
	OF	Y=2.394x-12.810	1830.639	1416.837~2331.562	0.9927	0.9032	1.69
	AI	Y=3.623x-11.217	1248.090	1032.429~1466.032	0.9878	1.3131	1.15
	SS	Y=3.355x-10.178	1081.002	886.868~1289.681	0.9837	1.5416	1.00
